# Unveiling the Status of Foot Self-Care Among Patients With Type 2 Diabetes in Western India: A Cross-Sectional Study

**DOI:** 10.7759/cureus.84312

**Published:** 2025-05-17

**Authors:** Sanjana Maniktalla, Prachee A Makashir, Jayashree Gothankar

**Affiliations:** 1 Community Medicine, Bharati Vidyapeeth (Deemed to be University) Medical College and Hospital, Pune, IND; 2 General Medicine, Bharati Vidyapeeth (Deemed to be University) Medical College and Hospital, Pune, IND

**Keywords:** diabetic foot care, foot hygiene, knowledge and practice, socio-demographic variables, type-ii diabetes mellitus

## Abstract

Background: Diabetic foot is a major complication of diabetes with a recurrence rate of 66% and an amputation rate of 12%. The first step in addressing the healthcare needs and necessary interventions for diabetic foot care is to assess the existing knowledge and practices among diabetic patients. With this objective, the study was conducted.

Methodology: A hospital-based cross-sectional study was conducted over six months. Pre-tested, validated, and structured questionnaire, containing sociodemographic, knowledge, and diabetic foot care practice domains, was administered to type 2 diabetes mellitus patients above the age of 30 years by interview technique. Spearman correlation and chi-square test were used for multivariate analysis. Results were reported with a 95% confidence interval (CI) and a statistically significant *P*-value of <0.05.

Results: The mean age of the patients was 51.12 ± 10.5 years. The sample included an equal proportion of males and females (50% each). Among the participants, 136 (82.42%) lacked adequate knowledge of foot hygiene, and 96% were unaware of how to perform self-examination of their feet at home.

Conclusions: The majority of patients had poor knowledge and practice of diabetic foot care. Hence, they’re more likely to end up with diabetic foot. This study emphasizes the preventable aspect of diabetic foot and how poor knowledge and practices can play a detrimental role in the occurrence of diabetic foot.

## Introduction

Currently, noncommunicable diseases are emerging as the new pandemic, with diabetes as a major component with a global burden of 422 million people, the majority of which is contributed by the low- and middle-income countries, and 1.5 million deaths annually [1,2]. In India, 77 million people aged 18 years and above are suffering from type 2 diabetes, and 25 million are prediabetics (at higher risk of developing diabetes shortly). More than 50% of the population is presently unaware of their diabetic status, which can greatly hamper their treatment efficacy and also increase their predisposition to the development of health complications if not detected and treated early [3-6]. Often asymptomatic, type 2 diabetic patients usually present with polyuria, polyphagia, polydipsia, and weight loss, with the development of complications, including diabetic nephropathy, retinopathy, neuropathy, etc. [5,7]. One major complication is diabetic foot, with an annual prevalence of almost 10% and an attributable risk of its incidence of almost 25% in diabetic patients [8]. Its risk factors can be divided into external and internal risk factors. External or controllable risk factors are modifiable and in control of the individual (minor and thermal trauma, smoking and alcohol consumption, inadequate control of blood sugar, obesity, and lack of patient cooperation). Internal or uncontrollable factors are non-modifiable and not in control of the individual suffering from diabetes (male gender, vasculopathy, age, duration of diabetes, and history of previous foot ulcers). By controlling external factors and targeting interventions toward modifying them, this complication of diabetic foot can be majorly prevented [9-11]. The development of a diabetic foot is usually due to peripheral neuropathy along with microvascular compromise, leading to blood stasis, necrosis, and gangrene. Even after successful management, the recurrence rate is 66%, and the amputation rate is 12% [12]. However, by providing appropriate foot care by comprehensive foot examinations by the physician, strict monitoring of blood sugar levels, and counselling the patient on diabetic foot ulcer, its etiology, self-examination of feet at home, wearing cotton socks, and padded shoes without front opening, maintaining appropriate hygiene, and reporting to the physician in case they observe even a small wound which is non-healing can create a huge difference in the outcome of this complication [13,14]. Hence, to gauge an idea of the necessary interventions, current knowledge, and practices need to be assessed in the patients, and appropriate counselling regards to health promotion and specific protection needs to be given to them to prevent this major complication of diabetes. Therefore, keeping the above factors and research gaps in mind, our objective of this study was to determine the knowledge and practices of footcare in type 2 diabetic patients in a tertiary care setting.

## Materials and methods

It was a hospital-based cross-sectional study conducted over six months. All consecutive patients from both the medicine and surgery outpatient and inpatient departments of the tertiary care hospital were selected. Inclusion criteria were decided as all adults aged 30 years and above diagnosed with type 2 diabetes for more than three months. To calculate sample size, prevalence of awareness of foot care to be 37% as seen in study of similar objective conducted by Taksande B in assessing knowledge, attitude, and practice of footcare in patients with diabetes at central rural India [15] was considered with absolute error of 20% and confidence level (CI) of 95%. According to this, the sample size was calculated to be 164 and rounded off to 165. Patients with a previous history of diabetic foot ulcer, type 1 diabetes mellitus, gestational diabetes mellitus, drug-induced diabetes, and immunocompromised diabetes were excluded from the study to prevent any possible bias or role of confounders in the analyzed results. The research tools used were a validated structured questionnaire filled out by the interview method. It consisted of three sections: Section A containing sociodemographic information, Section B containing information on diabetic status, and Section C containing 11 questions to test knowledge and 12 questions to check for practices regarding diabetic foot care, grouped under three sub-categories of maintaining foot hygiene, wearing appropriate footwear, and managing minor trauma to feet. An answer key was prepared and kept with the interviewer. Under the domain of knowledge, out of 11 questions, the maximum score was 1, and the minimum score was 0 for each question. The total knowledge score was 11, and the cutoff score was considered to be half of the total knowledge score, that is, 5.5. Participants scoring less than 5.5 were deemed to have poor knowledge, and those scoring more than or equal to 5.5 were considered to have adequate knowledge. Under the domain of practice, 12 questions were asked, each with a minimum score of 0 and a maximum of 1. The total practice score was 12, and the cutoff score was considered to be half of the total practice score, that is, 6. Those participants scoring less than 6 were considered to have poor practice, and those scoring equal to or more than 6 were considered to have good practice of diabetic footcare. Before commencing the study, approval was taken from the institutional ethics committee, the medical director of the tertiary care hospital, and the head of the department. A pilot study was conducted for pretesting of the questionnaire on 10%, i.e., 17 participants. The questionnaire was finalized based on observations from the pilot study, then translated into Hindi and back-translated into English to ensure accuracy. The questionnaire was validated by a subject expert, i.e., an endocrinologist. After taking prior written consent from the patients and explaining the objectives clearly, a questionnaire was administered by interview technique by the principal investigator. Questions based on sociodemographic, knowledge, and practice domains were asked, and the responses were scored accordingly. After doing so, the patients were counselled regarding appropriate foot care knowledge and self-footcare practices. 

Statistical analysis

Statistical analysis was done using SPSS Software (Version 28.0, IBM Corp., Armonk, NY). Continuous variable results were shown by descriptive statistics. Categorical variable results were shown by frequency and percentages. The chi-square test was used to test the association between sociodemographic variables and diabetic history with the domain of knowledge and practice. Pearson's correlation was used to detect a correlation between the knowledge and practice domains. Throughout the results, a 5% level of significance was used. All results are shown with a 95% CI, and a *P*-value <0.05 was considered significant.

Ethical statement

The study was conducted after obtaining permission from the institutional ethics committee (IEC) (BVDUMC/IEC/44).

## Results

The mean age of study participants was 51.12 ± 10.57 years. The study included an equal number of males and females, i.e., 50%, i.e., 83 males and 82 females (*N* = 165). Almost half, i.e., 49.9% of study participants were unemployed, and around 24% were graduates or above. Thirty-seven (23%) participants had acquired education up to the primary level (Table 1).

**Table 1:** Frequency distribution of sociodemographic characteristics (N = 165). Data represented as *N* and %. HSC, Higher Secondary Certificate; SSC, Secondary School Certificate

Sr. No.	Category		Frequency	%
1	Gender	Male	83	50.30
		Female	82	49.70
2	Occupation	Employed	84	50.91
		Unemployed	81	49.09
3	Education	Uneducated	31	18.79
		1st-4th standard	37	22.42
		5th-9th standard	26	15.76
		HSC+SSC	32	19.39
		≥Graduate	39	23.64

About the domain of duration of diabetes, 32.7% had diabetes for one to five years, and 23.63% had diabetes for 5-10 years.

**Figure 1:**
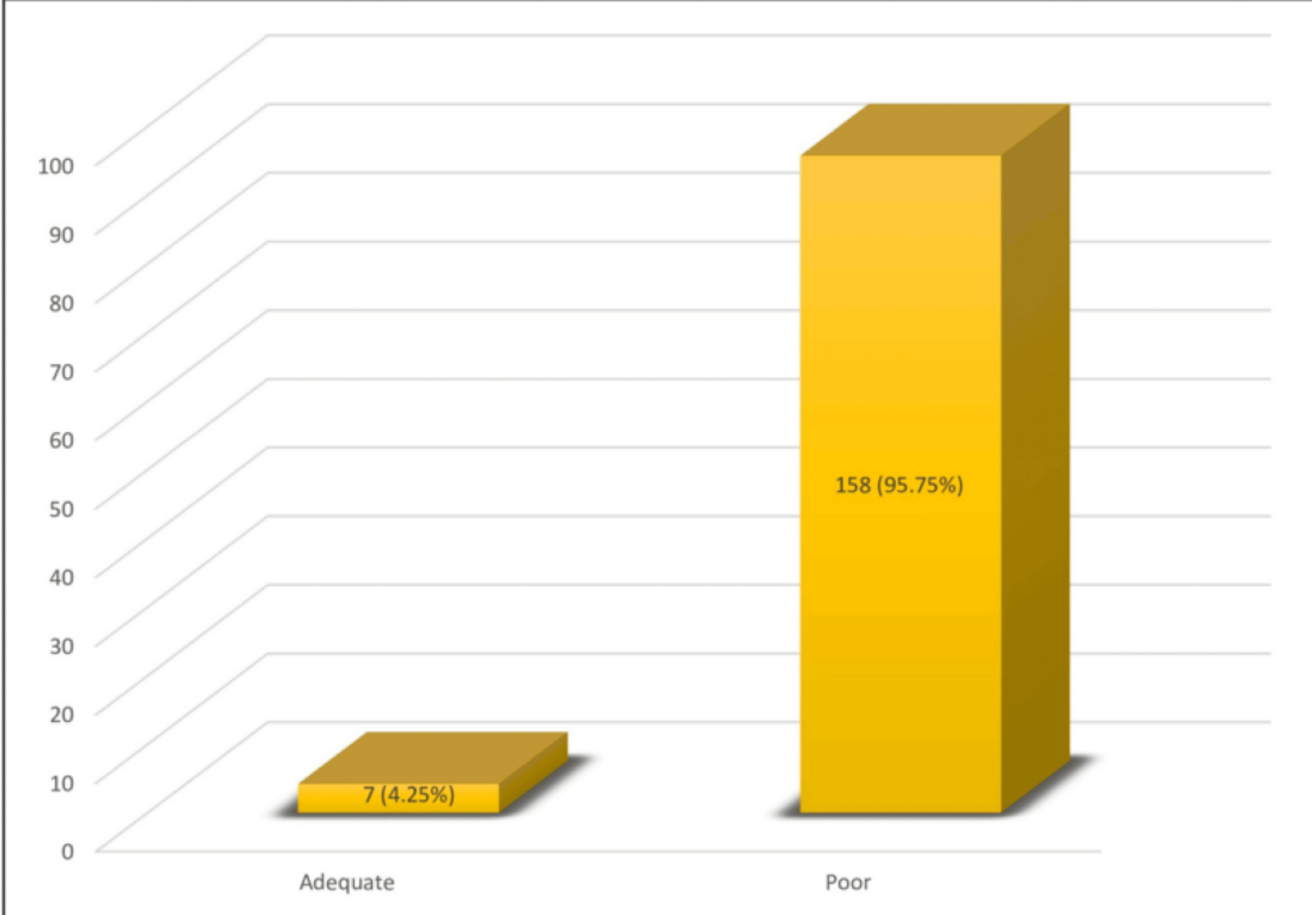
Bar graph showing participants’ knowledge of foot care among type 2 diabetic patients, presented as frequency and percentage (N = 165).

With regards to the knowledge domain, participants scoring less than half of the total score, which is 5.5 for the knowledge domain in the questionnaire, were deemed to have inadequate knowledge, and participants scoring equal to or more than half of the total score were considered to possess adequate knowledge of footcare. Inadequate knowledge regarding foot care in diabetes was observed in 158 (95.7%) participants (Figure 1).

**Figure 2:**
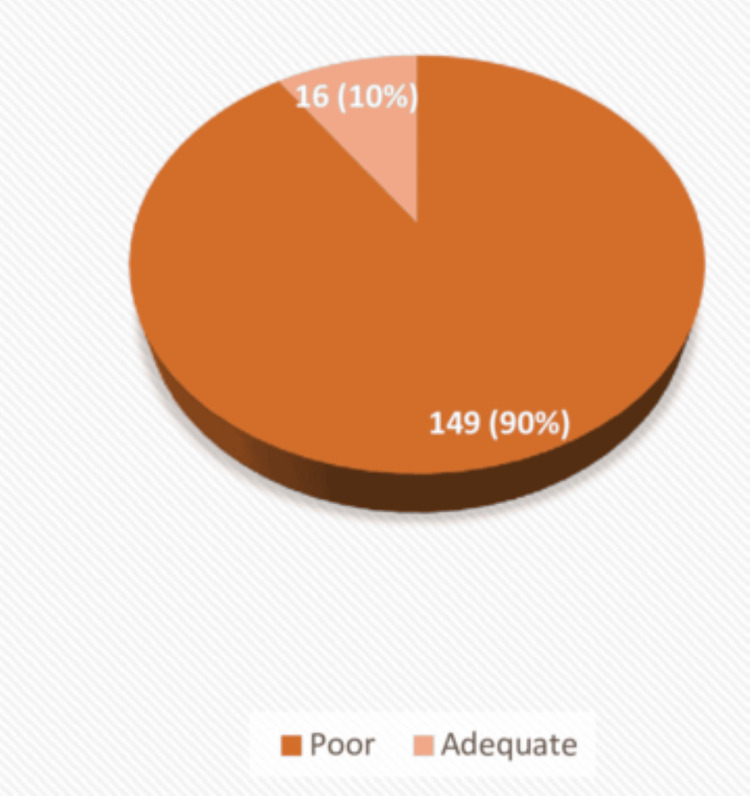
Pie chart depicting foot care practices among type 2 diabetic patients, shown as frequency and percentage (N = 165).

Similarly, participants scoring less than half of the total score, which is 6 in the practice domain in the questionnaire, were stated to have inadequate practice of footcare. Participants scoring equal to or more than half of the total score were considered to possess adequate practice of footcare. Thus, 149 (90.4%) had poor practice related to foot care (Figure 2). 

The knowledge and practice domains were divided into three sub-categories of foot hygiene, appropriate footwear, and minor trauma to feet. About maintenance of foot hygiene, within the knowledge domain, we observed that 92% of study participants had never heard about diabetic foot care, and 95.15% did not have any knowledge about self-examination of feet at home. By practices of the same sub-category, 92.12% did not practice appropriate methods to maintain foot hygiene. With regards to wearing appropriate footwear, almost 80.0% of study participants were aware that they should not walk barefoot inside or outside of their house; however, 73.3% did not know the type of footwear that should be worn to prevent the occurrence of diabetic ulcers. Similarly, in the domain of practice, 72% did not practice wearing soft padded shoes or shoes with front closure. By management of minor trauma to the foot, which included self-removal of calluses, tending to small wounds on the foot, and consulting their physicians, almost 50.0% of participants did not hold adequate knowledge of same, and about same sub-category in the practice domain, 63% had poor practice of all these factors (Table 2).

**Table 2:** Frequency and percentage distribution of knowledge and practice domains across different foot care subcategories (N = 165).

Sr. No	Sub-category	Domains			
		Knowledge		Practice	
		Inadequate, *N* (%)	Adequate, *N* (%)	Poor, *N* (%)	Good, *N* (%)
1	Maintaining feet hygiene	136 (82.42)	29 (17.57)	152 (92.12)	13 (7.87)
2	Appropriate footwear	119 (73.33)	46 (26.66)	119 (72.12)	46 (27.87)
3	Management of minor trauma	81 (49.09)	84 (50.9)	105 (63.63)	60 (36.36)

In the knowledge domain, the majority of the participants were in the age group of 40-65 years, out of which 94.7% had inadequate knowledge and 90.4% had poor practice of footcare. Under the category of gender, no major difference was seen in knowledge of foot care. Inadequate knowledge was observed in 95.1% of females and 96.4% of males. However, in the practice domain, 87.8% of females and 92.8% of males had poor practice of footcare. Regarding employment, out of 81 unemployed participants, 95.1% fell in the category of inadequate knowledge, while 88.9% had poor practices of footcare. Under the category of education, 37 participants had studied only till 4th standard, out of which 97.3% had inadequate knowledge and 89.2% had poor practice, being similar to 39 participants who attained education at a level equal or greater than that of a graduate, out of which 92.3% had inadequate knowledge and 84.6% had poor practice of footcare. With regards to association, none of the sociodemographic variables had a significant association with diabetes. However, about duration of diabetes was significantly associated (*P *< 0.05) with the domain of knowledge (Tables 3-4).

**Table 3:** Frequency distribution and association of sociodemographic variables with domain of knowledge using chi-square test (N = 165). *P*-value < 0.05 is significant.

Sr. No.	Variable	Category	Knowledge, *N *(%)		Total	Chi-square	*P*-value
			Adequate	Inadequate			
1	Age (years)	30-45	0 (0)	32 (100)	32		
		46-65	6 (5.3)	108 (94.7)	114		
		>65	1 (5.3)	18 (94.7)	19		
		Total	7 (4.2)	158 (95.8)	165 (100%)	1.759	0.415
2	Gender	Female	4 (4.9)	78 (95.1)	82		
		Male	3 (3.6)	80 (96.4)	83		
		Total	7 (4.2)	158 (95.8)	165 (100%)	0.162	0.687
3	Occupation	Unemployed	4 (4.9)	77 (95.1)	81		
		Employed	3 (3.6)	81 (96.4)	84		
		Total	7 (4.2)	158 (95.8)	165 (100%)	0.19	0.663
4	Education	Uneducated	2 (6.5)	29 (93.5)	31		
		1st-4th standard	1 (2.7)	36 (97.3)	37		
		5th-9th standard	0 (0.00)	26 (100)	26		
		HSC + SSC	1 (3.1)	31 (96.9)	32		
		≥Graduate	3 (7.7)	36 (92.3)	39		
		Total	7 (4.2)	158 (95.8)	165 (100%)	2.981	0.561

**Table 4:** Frequency distribution and association of sociodemographic variables with domain of practice assessed using the chi-square test (N = 165). *P*-value < 0.05 is considered significant.

Sr. No.	Variable	Category	Practice, *N* (%)		Total	Chi-square	*P*-value
			Good	Poor			
1	Age (Years)	30-45	4 (12.5)	28 (87.5)	32		
		46-65	11 (9.6)	103 (90.4)	114		
		>65	1 (5.3)	18 (94.7)	19		
		Total	16 (9.7)	149 (90.3)	165 (100%)	0.714	0.7
2	Gender	Female	10 (12.2)	72 (87.8)	82		
		Male	6 (7.2)	77 (92.8)	83		
		Total	16 (9.7)	149 (90.3)	165 (100%)	1.162	0.281
3	Occupation	Unemployed	9 (11.1)	72 (88.9)	81		
		Employed	7 (8.3)	77 (91.7)	84		
		Total	16 (9.7)	149 (90.3)	165 (100%)	0.363	0.547
4	Education	Uneducated	4 (12.9)	27 (87.1)	31		
		1st-4th standard	4 (10.8)	33 (89.2)	37		
		5th-9th standard	0 (0.00)	26 (100)	26		
		HSC + SSC	2 (6.3%)	30 (84.6%)	32		
		≥Graduate	6 (15.4%)	33 (84.6%)	39		
		Total	16 (9.7%)	149 (90.3%)	165 (100%)	5.083	0.279

With regards to correlation, a significant correlation was seen between knowledge and practice domains, falling under the category of moderate (correlation coefficient = 0.642) (Table 5).

**Table 5:** Pearson correlation to depict the correlation between knowledge and practice domains. *P*-value < 0.05 is considered significant.

		Knowledge score	Practice score
Knowledge score	Correlation coefficient	1.000	0.642^**^
	*P*-value		0.000
	N	165	165

## Discussion

In our study, the mean age was 51.12 ± 10.57 years. In studies conducted by Taksande et al. [15] and Ghasemi et al. [16] to assess diabetic foot care, a similar mean age was seen. However, a multi-center national survey study conducted by Dixit et al. in June 2022 [17] showed a higher mean age of 58.2 ± 8.6 years. In our study, no gender disparity was seen owing to a near equal selection of male and female study participants, similar to Ng et al.'s study [11]. However, in a study conducted by other Indian authors, in different study settings [17-19], a higher proportion of study participants were males, signifying a higher prevalence of diabetes and its stated complications in males as compared to females. About employment and education, almost 50.0% were unemployed, and 23% had studied till the primary level. In a study by George et al. in Southern India [19], 23.6% had studied till the primary level; however, only 0.9% were unemployed. Similarly, studies by Shaki et al. [20] and Shankhdar et al. [21] found that a low level of education and employment was found to have a significant impact on diabetic foot care knowledge and practices. This study showed that almost one-third of patients had diabetes for one to five years, similar to the study by George et al. [19] and Pourkazemi et al. [22]. However, studies by various other authors [3,23-24] showed a higher mean duration of diabetes of greater than or equal to 10 years, vastly higher than ours. This can be attributed to a different study and environmental setting, along with lifestyle choices and health-seeking behavior. The type of health services rendered also varies geospatially, which can contribute to the difference in the duration of diabetes. In our study, 95.7% of participants had inadequate knowledge regarding diabetic foot care. This can be attributed to a rush in OPDs, which makes it nearly impossible to educate each patient at length regarding foot care.

Another major factor is the increase in the national burden of diabetes, to almost 77 million, which further contributes to these complications. Studies done by numerous authors [9,12,21,25] showed a similar finding of poor knowledge regarding diabetic foot care, emphasizing the need to focus on knowledge awareness and self-examination for diabetic foot by *at-risk* individuals. Although other similar studies [15,23-24,26] showed that a minor proportion of their sample size only had poor knowledge, it can be attributed to a different research tool used as well as sampling technique, study setting, health-seeking behavior, and healthcare delivery system, all of which are crucial factors in diabetes management. In our study, almost 91% had poor practice of foot care. This can again be attributed to a lack of previous counseling as well as the efficiency on the part of patients in doing the same. Another study by Muduli et al. [12], Jayaprakash et al. [26], and Mahon et al. [14] showed poor practices of foot care in more than 60% of their study participants. However, previously quoted studies [15,23-24] all show good practice of footcare, pointing towards a correlation between poor knowledge and practice. In the sub-category of foot hygiene, which included questions on regular washing of feet, keeping the feet dry, cutting of toenails, and wearing clean socks, 82.42% of study participants were not aware of all these methods, and by practices of same sub-category, 92.12% did not practice these appropriate methods to maintain foot hygiene. A similar finding was seen in the study by Shaki et al. [20], almost 75% of study participants did not practice these aforementioned safe practices, especially not using nail cutters, but instead using scissors. Also, a study by Dhandapani et al. [23] showed that 48.4% did not know the proper technique to trim toenails. The same study showed that 84.2% of study participants did not know how to keep their feet dry, also seen in the study by Nance et al. [27]. In the sub-category of appropriate footwear, we saw a striking positive number in which 80% of participants were aware of not stepping outside barefoot. A similar study by Dixit et al. [17] showed a high percentage (60%) of not walking barefoot. However, wearing shoes with front closure, inspecting them before wearing them, and buying shoes that are soft and padded were not seen in the majority of participants, with almost 73% having inadequate knowledge and poor practice. Studies by various other authors [27,28-29,30] indicated the majority of participants with similar findings regarding walking barefoot, wearing inappropriate footwear, and neglecting to keep their feet dry, all aggregating to the gap in knowledge about foot hygiene in diabetic foot care.

In another sub-category of dealing with minor trauma to feet, such as self-removal of corns and calluses, consulting a physician in case of foot trauma, applying emollients to dry skin, and undergoing annual examinations, our study showed a lack of knowledge of almost 50% but a higher avoidance of these practices of almost 63%. In a study by Dixit et al. [17] and Alharbi et al. [24], almost 77% of participants never got their annual examinations or visited a doctor for minor wounds to their feet. Our study was unique in showing a detailed sociodemographic distribution of frequency within a specific category of these variables. For example, in the sub-category of employment, 81 unemployed participants, 95.1%, fell in the category of inadequate knowledge, while 88.9% had poor practice. Also, our study revealed insufficient knowledge to be equally distributed among males and females, directly contesting the results of other studies mentioned above, attributed to the uniqueness of this study. Our study showed no association between sociodemographic variables but a moderate positive correlation between knowledge and practice, similar to findings in above quoted studies [14,17,23,26]. These findings go in contrast with the findings of studies done by Pourkazemi et al. [22], where a significant association was seen between sociodemographic variables and knowledge and practice domain, owing to differences in the study setting, participants, and sociodemographic variables.

However, the latter study also showed a higher positive correlation between knowledge and practice, similar to our study. Duration of diabetes was found to be significantly associated (*P *< 0.05) with the domain of knowledge, as also seen in other studies [15,21,23]. This indicates that although the duration of diabetes is higher, knowledge about diabetic foot care is poor, indicating a major void in the services of our healthcare delivery system about preventive and health promotion medicine.

## Conclusions

Our study brings out the high knowledge and practice gap about diabetic foot care. It addresses the socio-demographic determinants in association with the knowledge and practice of foot care. It highlights the inadequacy of maintaining foot hygiene, wearing appropriate footwear, and managing wounds on the feet in the initial stages, which are essential components of foot care in diabetes. Our study highlights the less acknowledged preventive aspect of diabetic foot complications and the much-needed time-sensitive issue of effective counseling of diabetic patients.

To address the high knowledge and practice gap, there is a need to enforce targeted interventions like setting up a separate diabetes outpatient department that includes a physician and an endocrinologist. Educational interventions can include the provision of a counselor who advises on the prevention of complications associated with diabetes and the need for regular check-ups for prevention of diabetic foot and retinal, renal complications. This study stresses the urgency to address this void in our healthcare delivery system, identify the interluding factors, judiciously use resources, and provide interventions across all levels of healthcare to prevent complications like diabetic foot in the masses altogether.
